# The effects of individual nonheritable variation on fitness estimation and coexistence

**DOI:** 10.1002/ece3.5437

**Published:** 2019-08-01

**Authors:** M. Gabriela M. Gomes, Jessica G. King, Ana Nunes, Nick Colegrave, Ary A. Hoffmann

**Affiliations:** ^1^ Liverpool School of Tropical Medicine Liverpool UK; ^2^ CIBIO‐InBIO, Centro de Investigação em Biodiversidade e Recursos Genéticos CMUP, Centro de Matemática da Universidade do Porto Porto Portugal; ^3^ School of Biological Sciences, Institute of Evolutionary Biology University of Edinburgh Edinburgh UK; ^4^ Departamento de Física, Faculdade de Ciências BioISI – Biosystems and Integrative Sciences Institute, Universidade de Lisboa Lisboa Portugal; ^5^ School of BioSciences Bio21 Institute, University of Melbourne Melbourne Vic. Australia

**Keywords:** bacterial growth, coexistence, cohort selection, fitness estimation, nonheritable variation

## Abstract

Demographic theory and data have emphasized that nonheritable variation in individual frailty enables selection within cohorts, affecting the dynamics of a population while being invisible to its evolution. Here, we include the component of individual variation in longevity or viability which is nonheritable in simple bacterial growth models and explore its ecological and evolutionary impacts. First, we find that this variation produces consistent trends in longevity differences between bacterial genotypes when measured across stress gradients. Given that direct measurements of longevity are inevitably biased due to the presence of this variation and ongoing selection, we propose the use of the trend itself for obtaining more exact inferences of genotypic fitness. Second, we show how species or strain coexistence can be enabled by nonheritable variation in longevity or viability. These general conclusions are likely to extend beyond bacterial systems.

## 1 INTRODUCTION

Niche theories in ecology (Chesson, 2000; Grant, 1986; Tilman, 1982) and adaptive theories in evolutionary biology (McDonald & Kreitman, 1991) emphasize mean differences between species and genotypes, respectively, as key to their coexistence or fixation. Neutral theories in ecology (Bell, 2000; Caswell, 1976; Hubbell, 2001) contend that levels of biodiversity conform to models where individuals have equal fitness and random processes make community compositions inherently unstable while maintaining overall levels of diversity. Neutral theories in evolution (Kimura, 1983) also focus on the role of a random process (genetic drift) in maintaining variation instead of niche‐based fitness variation across genotypes. Both neutral and niche/adaptive theories tend to overlook the significance of variation at the individual level despite its role in early theory (MacArthur & Levins, 1967).

Recently, there has been a resurgence of interest in the impact of intraspecific variation in ecology (Des Roches et al., 2018; Hart, Schreiber, & Levine, 2016; Lichstein, Dushoff, Levin, & Pacala, 2007; Violle et al., 2012), and nonheritable intragenotypic variation in evolution (Shen, Pettersson, Rönnegård, & Carlborg, 2012; Steiner & Tuljapurkar, 2012). There is substantial evidence for nonheritable variation in traits driven particularly by inherently stochastic variation in life history components including individual variation in longevity (Hashimoto et al., 2016; Kiviet et al., 2014; Steiner & Tuljapurkar, 2012). This type of variation lends itself to selection within cohorts (Hartemink & Caswell, 2018; Kendall & Fox, 2002) and is likely to contribute substantially to phenomena such as the low heritability of fitness and the high diversity of genotypes and species. It may also result in spurious trends when fitness effects are measured across environments and give false indications of topical mechanisms such as adaptive phenotypic plasticity, bet‐hedging, and epistasis (Graves & Weinreich, 2017). However, the implications of this type of variation for many ecological and evolutionary processes have not yet been explored.

Here, we explore the ecological and evolutionary impacts of individual nonheritable variation in longevity or viability adopting bacterial systems. Section 2 introduces two basic models of bacterial growth which will be used to build study systems in subsequent sections. Section 3 describes the performance of the first model when an antibiotic stress is introduced and confirms its capability to reproduce realistic survival curves (e.g., as in Balaban, Merrin, Chait, Kowalik, & Leibler, 2004). Section 4 presents the central result that individual nonheritable variation in longevity produces consistent trends when relative fitness between bacterial genotypes is measured across stress gradients. Finally, this leads to a hypothesis that this variation might stabilize coexistence, which is confirmed in Section 5 for the two model systems. These results are discussed more generally in Section 6.

## 2 BASIC MODELS

Supported by evidence from bacterial systems (Balaban et al., 2004; Cadena, Fortune, & Flynn, 2017; Gomes et al., 2019; Hashimoto et al., 2016; Jouvet, Rodriguez‐Rojas, & Steiner, 2018; Kiviet et al., 2014; Levin, 2004; Trauer et al., 2019), we build two model suites which in later sections will be used to explore how nonheritable variation in fitness components may affect the response of a population under different levels of stress, bias common measures of relative fitness between genotypes or strains and associated selection coefficients, and affect their ability to coexist when placed in competition for shared resources.

### 2.1 Bacterial growth models

**Figure 1:**
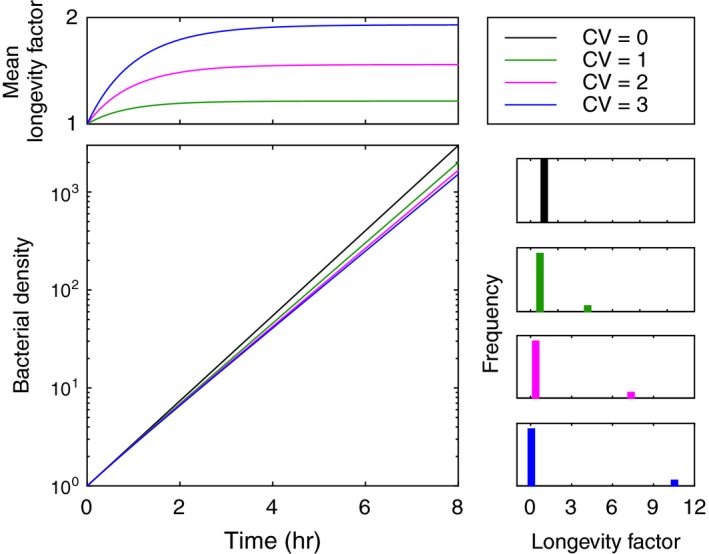
Bacterial growth with nonheritable variation in cell longevity. Solutions of model (1) with distributed longevity factors, γ, with mean $〈\gamma 〉=1$. The fraction of cell births entering the high‐longevity group was set to 0.09. Three distinct coefficients of variation are represented: CV=0 (black), CV=1 (green), CV=2 (magenta), and CV=3 (blue). Mean growth rates at birth are set to M=1 in all cases. This condition is also imposed at the beginning of all trajectories by setting initiation conditions accordingly: $B_{i}\left(0\right)=p_{i}$, for i=1,2. Growth curves bend due to the accumulation of long‐lived cells, and this effect increases with CV

First, we consider bacteria growing under in vitro laboratory conditions with nonheritable variation in cell longevity (elapsed time between cell birth and division) (Hashimoto et al., 2016; Jouvet et al., 2018; Kiviet et al., 2014; Powell, 1958). To facilitate specific arguments to be made about mother and daughter cells, in our models we separate the process of cell division into death of mother cells and birth of daughter cells. In the simplest instance of a single genotype with unlimited resources, this is written as follows:

(1) $$\frac{dB_{i}}{\mathrm{dt}}=p_{i}\beta \sum_{j=1}^{n}\frac{B_{j}}{\gamma_{j}}-\frac{\mu }{\gamma_{i}}B_{i},$$

where *B_i_*, for i=1,…,n, denote the concentration of bacteria with longevity factor $\gamma_{i}$, in a fraction $p_{i}$ of all births, purporting a distribution with mean $〈\gamma 〉=\sum_{i}p_{i}\gamma_{i}$, variance $〈{\left(\gamma -〈\gamma 〉\right)}^{2}〉=\sum_{i}p_{i}{\left(\gamma_{i}-〈\gamma 〉\right)}^{2}$, and coefficient of variation $\text{CV}=\sqrt{〈{\left(\gamma -〈\gamma 〉\right)}^{2}〉}/〈\gamma 〉$ treated as a varying parameter. Parameter μ controls the mean rate of cell division which, to enable fitness comparisons across distributions, we normalize such that $M=〈\mu /\gamma 〉=1$. Considering that cells replicate by binary fission, we impose β=2μ (i.e., cells are born at twice the rate that they cease to exist). Figure 1 depicts typical growth curves generated by this model, together with mean cell longevity factors which effectively increase from a common initial value ($〈\gamma 〉=1$ in all cases) as longer‐lived cells accumulate (selection for higher longevity and reduced growth). Instantaneous growth rates converge to purely exponential, but the asymptotic limits are lower for higher coefficients of variation even though all populations have their growth distributions being constantly reset to the same mean through births (variation in individual longevity is nonheritable). Without variation (CV=0) selection vanishes and the model defaults to exact exponential growth (dB/dt=μB, where $B=\sum_{i}B_{i}$), but any arbitrarily small perturbation that confers nonheritable variation in cell longevity will induce the phenomenon described here and set the scene for a multitude of outcomes which we describe in subsequent sections.

As noted by Hashimoto et al. (2016), nonheritable variation in longevity can also reduce the doubling time of a population in relation to the mean longevity of its constitutive cells. Simple arguments, which attend to the normalization M=1, show that this finding is compatible with our results (not shown).

We note, however, that not all fitness traits lend themselves to this form of selection when variation is nonheritable. Nonheritable variation in fecundity, for example, may also result in reduced growth but, in contrast with the description above, this is due to stochastic effects which become negligible in large populations (Gillespie, 1974).

### 2.2 Host colonization models

**Figure 2:**
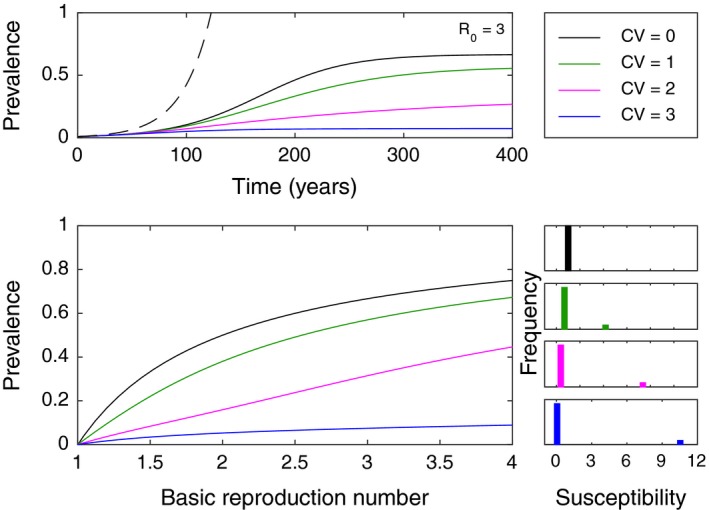
Prevalence of a bacterial strain facing variation in individual host susceptibility. Solutions of model (2)–(3) with distributed susceptibility factors, α, as a function of the basic reproduction number $R_{0}=〈\alpha 〉\beta /\mu$, assuming mean $〈\alpha 〉=1$. The fraction of high‐susceptibility hosts was set to 0.09. Four distinct coefficients of variation are represented: CV=0 (black), CV=1 (green), CV=2 (magenta), and CV=3 (blue). The dashed curve represents exact exponential growth with unlimited uniform hosts (dI/dt=βI) for comparison. Other parameters: μ=1/80 per year. Limited resources limit growth, naturally, and variance in host susceptibility leads to lower colonization prevalence due to the accumulation of less suitable hosts in the susceptible pool

Second, we resort to models for microbial colonization of a host population to address variation in susceptibility among hosts (Diekmann, Heesterbeek, & Britton, 2012; Gomes et al., 2019) as a natural manifestation of variation in resource suitability (from the bacterial viewpoint):

(2) $$\frac{dS_{i}}{\mathrm{dt}}=p_{i}\mu -\alpha_{i}\beta IS_{i}-\mu S_{i}$$

(3) $$\frac{\mathrm{dI}}{\mathrm{dt}}=\sum_{i=1}^{n}\alpha_{i}\beta IS_{i}-\mu I,$$

where μ is the host death and birth rate, β is the effective contact rate between infective (colonized) and susceptible hosts, $\alpha_{i}$ is the susceptibility factor of hosts $S_{i}$ that enter the system as a fraction $p_{i}$ of all births, purporting a distribution with mean $〈\alpha 〉$, variance $〈{\left(\alpha -〈\alpha 〉\right)}^{2}〉$, and coefficient of variation $\text{CV}=\sqrt{〈{\left(\alpha -〈\alpha 〉\right)}^{2}〉}/〈\alpha 〉$ treated as a varying parameter. In line with the previous system, also in this case a form of nonheritable variation reduces bacterial growth, now manifested as fewer hosts being colonized (Figure 2). When four bacterial strains with the same infectivity (i.e., same $〈\alpha 〉$) independently invade a host population, the growth curves for the prevalence of colonized hosts start tangential to the same exponential growth curve and decelerate to reach an equilibrium as susceptible hosts are depleted. One of the strains finds all hosts equality suitable and as a result experiences the minimal deceleration and reaches the highest endemic prevalence. The other strains find some hosts more suitable than others, decelerate more due to the accumulation of less suitable hosts in the susceptible pool and reach endemic equilibria which are lower for higher variances in host susceptibility.

In the treatment below, we build more elaborate systems from the blocks introduced here, always considering that nonheritable variation in fitness is affected by selection even though measurements of selection coefficients typically focus on only heritable components of variation (Chevin, 2011). Analyses are presented incrementally, with various results being highlighted along the way, concluding with an exposition of how coexistence of bacterial genotypes or strains can be maintained by nonheritable variation in individual fitness components. The mechanisms rely on mean‐variance trade‐offs which can be arbitrarily small leading us to note the fragility of strict neutrality formulations and adding to already expressed concerns about their merits as null hypotheses (Gotelli & McGill, 2006).

## 3 NONHERITABLE VARIATION UNDER ANTIBIOTIC STRESS

Populations of genetically identical bacteria placed under selective antibiotic pressure typically exhibit a decline over time in their rates of mortality (Balaban et al., 2004; Levin, 2004). When observed in time frames that are not long enough to reflect increases in the frequency of heritable mutations, this pattern has been attributed to nonheritable variation in sensitivity of individual cells to the antibiotic, which in turn has been linked to variation in rates of cell growth and division. The notion that individual bacterial cells of the same genotype vary in their rates of cell division is supported by independent studies that used microfluidic techniques to track thousands of bacterial cells to determine their individual lifespan and map division events (Hashimoto et al., 2016; Jouvet et al., 2018). Jouvet et al. (2018) concluded that 90% of the variability is nonheritable, presumably corresponding to the characteristics of each individual cell being molded by its own sequence of stochastic events throughout life.

To explore the impact of this nonheritable variation on the population response to antibiotics, we modify established mathematical formalisms representing bacterial population dynamics (Hsu, Hubbell, & Waltman, 1977; Smith, 1981; Stewart & Levin, 1973) to include individual variation in rates of cell division, similarly to how frailty variation has been treated in demography (Vaupel, Manton, & Stallard, 1979; Vaupel & Yashin, 1985). More specifically, we adopt model (1) and introduce an antibiotic that reduces the viability of newborn cells by a factor $\sigma_{a}$:

(4) $$\frac{dB_{i}}{\mathrm{dt}}=p_{i}\beta \left(1-\sigma_{a}\right)\sum_{j=1}^{n}\frac{B_{j}}{\gamma_{j}}-\frac{\mu }{\gamma_{i}}B_{i}.$$

**Figure 3:**
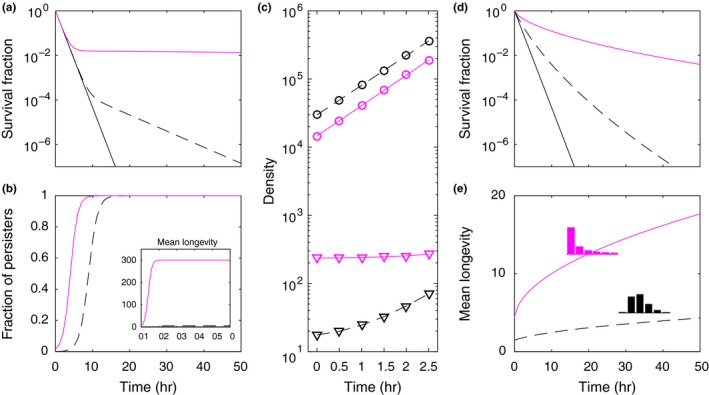
Bacterial persistence to antibiotic treatments. (a–c) Solutions of model (5)–(6) with two‐group distributed longevity factors, γ (Methods). The fraction of cell births entering the high‐longevity group was set to 0.0001. Two distinct coefficients of variation are represented: CV=0.05 (dashed black), and CV=3 (magenta). The solid black curve represents a homogeneous population: CV=0. A pre‐antibiotic phase ($\sigma_{a}=0$) was simulated with c=2 and ρ=0.003, until a stationary phase was established. Stationary phase solutions were used to simulate: (a, b), antibiotic introduction by setting $\sigma_{a}=0.9$ and turning off the chemostat flow (ρ=0); and (c), growth without antibiotic by keeping $\sigma_{a}=0$ and setting $R\left(0\right)=10^{6}$. Curves punctuated by circles represent total populations, whereas triangles refer to persistent fractions. (d, e), Solutions of model (5)–(6) with gamma distributed longevity factors and two distinct coefficients of variation: CV=0.5 (dashed black), and CV=2 (magenta). Other parameters: M=1, $\phi \left(R\right)=R/\left(1+R\right)$

Balaban et al. (2004) investigated the persistence of inherently sensitive cells when a population of genetically identical bacteria is exposed to an antibiotic stress, a phenomenon first observed in the early days of penicillin use (Bigger, 1944). The authors described mathematically the dynamics of surviving cells by switching mechanisms between a majority of rapidly growing (normal) cells and a minority of slowly growing (persister) cells. Coupling model (4) to a system of continuous resource provision we obtain:

(5) $$\frac{dB_{i}}{\mathrm{dt}}=\phi \left(R\right)p_{i}\beta \left(1-\sigma_{a}\right)\sum_{j=1}^{n}\frac{B_{j}}{\gamma_{j}}-\frac{\mu }{\gamma_{i}}B_{i}-\rho B_{i}$$

(6) $$\frac{\mathrm{dR}}{\mathrm{dt}}=\rho \left(c-R\right)-\phi \left(R\right)\beta \left(1-\sigma_{a}\right)\sum_{j=1}^{n}\frac{B_{j}}{\gamma_{j}},$$

where *R* is the concentration of resources in a chemostat, *c* its concentration in the input flow, ρ the rate at which medium enters and leaves the chemostat, and $\phi \left(R\right)=R/\left(1+R\right)$ is a nonnegative increasing function between 0 and 1 describing the viability of newly born cells as a function of resource availability. This parsimonious model is capable of reproducing the results of Balaban et al. (2004) without invoking phenotypic switches (Figure 3) and will be used for multiple purposes throughout this paper.

The model was solved numerically without antibiotic ($\sigma_{a}=0$) until reaching a stationary state, at which stage the antibiotic was introduced ($\sigma_{a}=0.9$). In the absence of variation in cell longevity, the antibiotic causes an exponential decay in cell density (solid black line in Figure 3a). The slightest variation in longevity induces a form of selection that results in decelerated population decay (illustrated by the dashed black line in Figure 3a generated with a coefficient of variation of 0.05). The greater the variation, the greater the deceleration (magenta line in Figure 3a generated with the coefficient of variation set to 3). Figure 3b shows the action of selection on cell longevity. As time under antibiotic increases, the faster dividing cells become rarer in the population (i.e., the fraction of persisters increases). The original distribution of cell longevity factors is continuously being reset through new births, but viability is generally low due to antibiotic pressure leading to an accumulation of long‐lived cells.

The same phenomenon occurs regardless of whether the population is structured into two discrete groups or shows a more continuous distribution of longevity factors (Figure 3d,e). Indeed, different types of survival curves, as reported by Balaban et al. (2004), can be obtained by concordantly setting the distribution of longevity factors without needing additional switches or other processes (Gefen, Gabay, Mumcuoglu, Engel, & Balaban, 2008; Johnson & Levin, 2013; Rotem et al., 2010). These ideas apply to growing cell populations more generally and may be extended to describe failure of treatments in cancer patients (Mizrahi, Gefen, Simon, & Balaban, 2016), as well as a wide variety of bet‐hedging strategies in nature (Philippi & Seger, 1989).

In contrast with adaptive phenotypic plasticity theory (Chevin, Lande, & Mace, 2010; Coulson et al., 2017; Pigliucci, 2001), our model populations are essentially the same irrespective of what stresses they may experience, but the nonheritable variation in a trait that affects fitness produces survival profiles dependent on environmental specificities. As a result, a population is never completely represented by those individuals who are alive at any one time and the exact misrepresentation depends on the nature and strength of environmental stresses.

## 4 RELATIVE FITNESS DEFINED ALONG STRESS GRADIENTS

**Figure 4:**
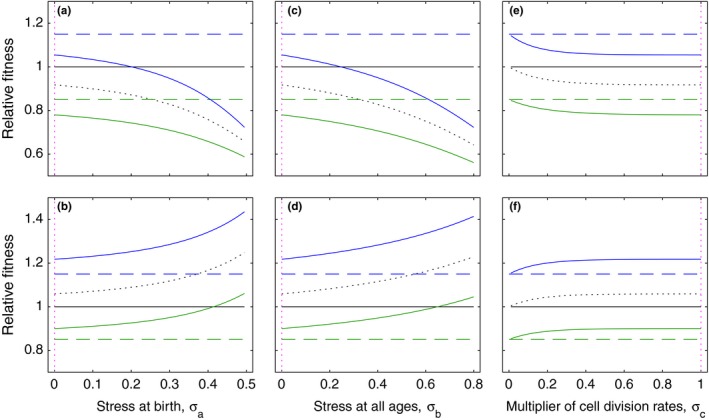
Relative fitness across stress gradients. Relative growth rates between mutant and ancestral genotypes calculated at time t=6h with: (a, c, e) M=1 and CV=0 (black, ancestral genotype), M=0.85 and CV=3 (green, mutant), M=1.15 and CV=3 (blue, mutant), and M=1 and CV=3 (black, dotted); (c, d, f) M=1 and CV=1 (black), M=0.85 and CV=0 (green), M=1.15 and CV=0 (blue), and M=1 and CV=0 (black, dotted). Stress was implemented in three ways: (a, b) reduction in cell viability at birth (parameter $\sigma_{a}$ in model (4)); (c, d) increase in cell mortality at all ages (parameter $\sigma_{b}$ in model (7)); or (e, f) factor affecting the rate of cell division (parameter $\sigma_{c}$ in model (8)). Vertical dotted lines (magenta) indicate where the three axes ($\sigma_{a}$, $\sigma_{b}$, $\sigma_{c}$) intersect. The fraction of cell births entering high‐longevity groups is set to 0.09

Fundamental to the results above is a notion of genotype fitness that is wider than that commonly used. By accommodating explicitly for individual nonheritable variation in longevity, the measurable genotype fitness becomes dependent on the strength of selection which may vary between environments. We consider different intensities of environmental stresses which either act to reduce cell viability at birth ($\sigma_{a}$ in model (4)) (Figure 4a,b) or, alternatively, reduce survival at any age ($\sigma_{b}$):

(7) $$\frac{dB_{i}}{\mathrm{dt}}=p_{i}\beta \sum_{j=1}^{n}\frac{B_{j}}{\gamma_{j}}-\frac{\mu \left(1+\sigma_{b}\right)}{\gamma_{i}}B_{i}$$

(Figure 4c,d), or even act as a favorable factor reducing the need for cells to divide and thereby slowing down the rate of cell division ($\sigma_{c}$):

(8) $$\frac{dB_{i}}{\mathrm{dt}}=\sigma_{c}\left(p_{i}\beta \sum_{j=1}^{n}\frac{B_{j}}{\gamma_{j}}-\frac{\mu }{\gamma_{i}}B_{i}\right)$$

(Figure 4e,f). Considering mutants derived from ancestral genotypes, we describe the possible patterns which may occur when fitness ratios are measured ($r_{m}/r_{a}$, where $r_{a}$ and $r_{m}$ denote ancestor and mutant growth rates, respectively, as given by the right‐hand side of the respective model equations at some time point during exponential phase [illustrated at 6 hr in the figure but the results are not specific to this particular choice]). First, we assume that the phenotypic variance of the ancestor is negligible compared to the mutant and find this to result in measurable fitness ratios (solid colored lines in Figure 4a,c,e) that are consistently lower than those that would have resulted from the same mean effects if mutant and ancestor had the same variance (dashed lines), a discrepancy that increases with stress. This trend is common in data (Kraemer, Morgan, Ness, Keightley, & Colegrave, 2016), but the reverse has also been observed (Kishony & Leibler, 2003) and occurs in our framework when mutants are less variable than their ancestors (Figure 4b,d,f). The level of nonheritable variation therefore defines the relative fitness of a genotype across a gradient.

Genetic stresses can induce similar phenomena on new mutations and affect measurements of epistasis (Agrawal & Whitlock, 2010). Any mutation with an effect on fitness sets a differential in stress levels between ancestral and mutant genotypes, introducing a bias in the assessment of the effects of additional mutations. If an initial mutation has increased fitness variance, for instance, a second mutation may appear less deleterious without necessarily involving epistasis between the mutations. More generally, these trends may impact the estimation of distributions of fitness effects of mutations (Eyre‐Walker & Keightley, 2007; Perfeito, Fernandes, Mota, & Gordo, 2007; Robert et al., 2018).

In light of these issues, we argue that when nonheritable variation in individual fitness exists, unbiased fitness ratios between genotypes and corresponding selection coefficients ($1-r_{m}/r_{a}$) cannot be measured directly from population‐level observations but can be estimated by fitting a curve to measurements taken across stress gradients. The performance of a genotype is expected to vary along the gradient in response to the level of nonheritable variation present generally and specific to that genotype.

Common procedures for measuring fitness and associated quantities do not accommodate the phenomena described above. This is strikingly conveyed by Figure 4 where fitness curves of two genotypes measured across a stress gradient effectively cross at some critical stress value (solid blue and black curves in Figure 4a,c, and green and black curves in Figure 4b,d) where the selection coefficient appears to be zero. The populations differ, however, in their fitness distributions, and the crossing is due to the action of selection on nonheritable fitness components. This suggests that unaccounted nonheritable phenotypic variation within genotypes is capable of promoting coexistence or at least persistence of multiple genotypes and unexpectedly affect patterns of genetic variation (Johnson & Barton, 2005).

These considerations have implications for genetic variation within populations. Firstly, genetic diversity of fitness traits could be present but be difficult to detect, even though these traits typically have low heritability (Fisher, 1930; Merilä & Sheldon, 1999). Secondly, the effects of genetic drift on fitness traits may be slowed relative to models that account for fluctuations of selection over time (due to random environmental conditions) but no individual variation (Gillespie, 1973). Individual variation in longevity, as considered in our models, may also increase the persistence time of finite populations independently of environmental stochasticity (Kendall & Fox, 2002), with implications for the extent of adaptive evolution inferred from observations.

## 5 NONHERITABLE VARIATION PROMOTES STABLE COEXISTENCE

### 5.1 Bacterial growth models

A classic debate in community ecology concerns whether the high diversity of species able to coexist in competition for the same resources is attributed to “equalizing” (neutral theory) or “stabilizing” (niche theory) mechanisms (Chesson, 2000). The neutral theory (Hubbell, 2001) posits that individuals, irrespective of species, are basically identical in their fitness and their interactions, and community dynamics are driven by demographic stochasticity and speciation. The niche theory, by contrast, proposes that species differ in their niches (Grant, 1986; Tilman, 1982) and that the negative effects of intraspecific individual interactions are larger than those due to interspecific interactions. This dichotomy has also been presented as a contention between stochasticity and determinism (Chave, 2004). More recently, these arguments have relaxed considerably, mainly due to the increasing recognition of the significance of individual variation (Des Roches et al., 2018; Hart et al., 2016; Lichstein et al., 2007; Violle et al., 2012) to species coexistence.

**Figure 5:**
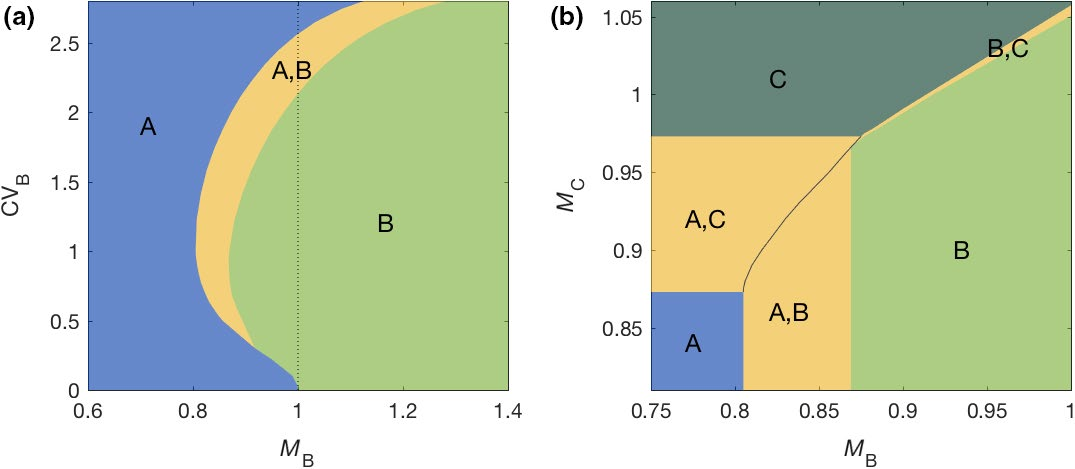
Stable coexistence of microbial species in an oscillating chemostat. Model (9)–(12) was solved numerically with two (a) and three (b) species (Methods). Yellow tongues represent regions of stable coexistence among the indicated species. All species have the same cell viability function $\phi \left(R\right)=R/\left(1+R\right)$, the chemostat flow is set to ρ=0.1, and the concentration of resources in the input flow oscillates as $c\left(t\right)=3\left[1+\cos\left(2\pi t/24\right)\right]$. Other parameters: (a, b) ${\text{CV}}_{A}=0$ and $M_{A}=1$; (b) ${\text{CV}}_{B}=1$ and ${\text{CV}}_{C}=2$

To add to this issue, we extend the models used above to accommodate two bacterial species (A homogeneous and B with variation in longevity) and introduce a 24 hr oscillation in the concentration of the single resource entering the system:

(9) $$\frac{\mathrm{dA}}{\mathrm{dt}}=\phi \left(R\right)\beta_{A}A-\mu_{A}A-\rho A$$

(10) $$\frac{dB_{i}}{\mathrm{dt}}=\phi \left(R\right)p_{i}\beta_{B}\sum_{j=1}^{n}\frac{B_{j}}{\gamma_{\mathrm{Bj}}}-\frac{\mu_{B}}{\gamma_{\mathrm{Bi}}}B_{i}-\rho B_{i}$$

(11) $$\frac{\mathrm{dR}}{\mathrm{dt}}=\rho \left[c\left(t\right)-R\right]-\phi \left(R\right)\left(\beta_{A}A+\beta_{B}\sum_{j=1}^{n}\frac{B_{j}}{\gamma_{\mathrm{Bj}}}\right),$$

where $c\left(t\right)=c_{0}\left[1+\cos\left(2\pi t/24\right)\right]$. Figure 5 shows a tongue‐shaped region (in yellow) outlining stable coexistence of the two species. Previous studies have described coexistence in similar systems (Hsu et al., 1977; Smith, 1981; Stewart & Levin, 1973), but relied on different species having different viability functions $\phi \left(R\right)$, thus complying strictly with the niche theory. In contrast, the mechanism we describe here relies on selection acting on individual variation in longevity under oscillating resources and would appear neutral if framed within traditional theories which are essentially blind to intraspecific individual variation. When resources are low, high‐longevity cells are at an advantage and so are species exhibiting higher variance, whereas under abundant resources species with lower variance have the advantage because they effectively grow faster, generating a pattern that can be interpreted as negative frequency‐dependent selection when no such dependence has been imposed. It has previously been noted that a similar mean‐variance trade‐off mechanism could stabilize coexistence in a plant system (Lichstein et al., 2007), although the effect was weak as it lacked the oscillation in resource availability.

Extending the model to three species, this mechanism does not appear to sustain coexistence of more than two species in our numerical explorations (Figure 5c), but fitness is typically governed by many traits and variation in other processes may conceivably extend possibilities for coexistence.

### 5.2 Host colonization models

Shifting from longevity to resource accessibility, and its effect on bacterial cell viability, we now build on model (2)–(3) for the colonization of a host population by multiple microbial strains, each affected by an independent distribution of host suitabilities (susceptibilities from the host viewpoint). The model for three strains (A, B, and C) circulating in a host population, with $n_{A}$, $n_{B,}$ and $n_{C}$ susceptibility groups, respectively, to each species is written as follows:

(12) $$\frac{dS_{i_{A}i_{B}i_{C}}}{\mathrm{dt}}=p_{Ai_{A}}p_{Bi_{B}}p_{Ci_{C}}\mu -\left(\alpha_{Ai_{A}}\beta_{A}I_{A}+\alpha_{Bi_{B}}\beta_{B}I_{B}+\alpha_{Ci_{C}}\beta_{C}I_{C}\right)S_{i_{A}i_{B}i_{C}}-\mu S_{i_{A}i_{B}i_{C}}$$

(13) $$\frac{dI_{A}}{\mathrm{dt}}=\sum_{i_{A}=1}^{n_{A}}\alpha_{Ai_{A}}\beta_{A}I_{A}\sum_{i_{B}=1}^{n_{B}}\sum_{i_{C}=1}^{n_{C}}S_{i_{A}i_{B}i_{C}}-\mu I_{A}$$

(14) $$\frac{dI_{B}}{\mathrm{dt}}=\sum_{i_{B}=1}^{n_{B}}\alpha_{Bi_{B}}\beta_{B}I_{B}\sum_{i_{A}=1}^{n_{A}}\sum_{i_{C}=1}^{n_{C}}S_{i_{A}i_{B}i_{C}}-\mu I_{B}$$

(15) $$\frac{dI_{C}}{\mathrm{dt}}=\sum_{i_{C}=1}^{n_{C}}\alpha_{Ci_{C}}\beta_{C}I_{C}\sum_{i_{A}=1}^{n_{A}}\sum_{i_{B}=1}^{n_{B}}S_{i_{A}i_{B}i_{C}}-\mu I_{C},$$

where $\beta_{X}$, for X=A,B,C, is the effective contact rate between hosts infective with strain X and susceptible hosts, $\alpha_{Xi_{X}}$, for $i_{X}=1,\text{…},n_{X}$, are the susceptibility factors of hosts $S_{...i_{X}...}$, who enter the system as fractions $p_{Xi_{X}}$ of all births, purporting distributions with mean ${〈\alpha 〉}_{X}=\sum_{i_{X}}p_{Xi_{X}}\alpha_{Xi_{X}}=1$, variance $〈{\left(\alpha_{X}-1\right)}^{2}〉=\sum_{i_{X}}p_{Xi_{X}}{\left(\alpha_{Xi_{X}}-1\right)}^{2}$, and coefficients of variation ${\text{CV}}_{X}=\sqrt{〈{\left(\alpha_{X}-1\right)}^{2}〉}$ treated as varying parameters. The strain‐specific basic reproduction numbers are $R_{0X}=\beta_{X}/\mu$.

In the special case where the host population is homogeneously susceptible to A ($n_{A}=1$), heterogeneous to B with two susceptibility groups ($n_{B}=2$), and C is absent ($n_{C}=0$), coexistence occurs when $R_{0A},R_{0B}>1$ and $\frac{-2\alpha_{B1}\alpha_{B2}R_{0B}}{-\alpha_{B1}\alpha_{B2}R_{0B}-\alpha_{B1}-\alpha_{B2}+\sqrt{{\left(\alpha_{B1}+\alpha_{B2}-\alpha_{B1}\alpha_{B2}R_{0B}\right)}^{2}-4\alpha_{B1}\alpha_{B2}\left(1-R_{0B}\right)}}<R_{0A}<R_{0B}.$

**Figure 6:**
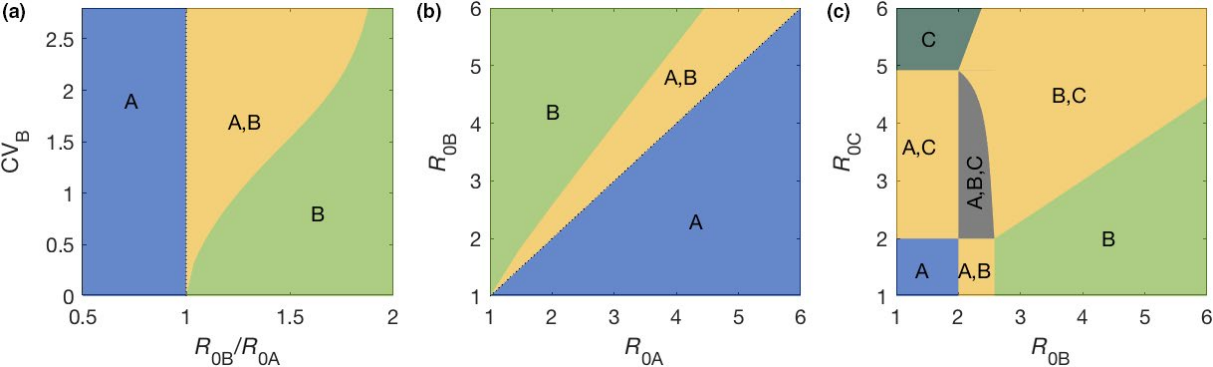
Stable coexistence of microbial species colonizing a host population. Model (13)–(16) was solved analytically with two (a, b) and three (c) species (Methods). Yellow regions represent conditions for two‐species stable coexistence as indicated, while three‐species coexistence is found in the gray zone (c). Other parameters: (a–c) ${\text{CV}}_{A}=0$; (b, c) ${\text{CV}}_{B}=1$; (c) ${\text{CV}}_{C}=2$ and $R_{0A}=2$

These conditions were used to delineate the two‐strain coexistence tongues in Figure 6a,b. In the case of three strains ($n_{C}=2$), the conditions for coexistence among the various pairs are analogous and were used to partially generate Figure 6c. To complete the figure, the three‐strain coexistence region, which exists for $R_{0A},R_{0B},R_{0C}>1$, is bounded by the straight lines $R_{0B}=R_{0A}$ and $R_{0C}=R_{0A}$ (where strains B and C, respectively, become absent), and by a third line, where strain A becomes absent. This line can be obtained analytically by assuming three‐strain coexistence and then setting the equilibrium abundance of strain A equal to zero.

The extension of the model to *N* strains is straightforward although the notation becomes dense:

(16) $$\frac{dS_{i_{1}i_{2}\text{⋯}i_{N}}}{\mathrm{dt}}=\prod_{X=1}^{N}p_{Xi_{X}}\mu -\sum_{X=1}^{N}\alpha_{Xi_{X}}\beta_{X}I_{X}S_{i_{1}i_{2}\text{⋯}i_{N}}-\mu S_{i_{1}i_{2}\text{⋯}i_{N}}$$

(17) $$\frac{dI_{X}}{\mathrm{dt}}=\sum_{i_{X}=1}^{n}\alpha_{Xi_{X}}\beta_{X}I_{X}\sum_{i_{1}=1}^{n}\text{⋯}\sum_{i_{X-1}=1}^{n}\sum_{i_{X+1}=1}^{n}\text{⋯}\sum_{i_{N}=1}^{n}S_{i_{1}i_{2}\text{⋯}i_{N}}-\mu I_{X}$$

where $\beta_{X}$, for X=1,…,N is the effective contact rate between hosts infective with strain X and susceptible hosts and all the remaining parameters are as before. In the special case where the host population is homogeneously susceptible to strain 1, we find an *N*‐strain coexistence region with all $R_{0X}>1$. This region has a simple geometry in the $R_{0X}$ space that generalizes the three‐strain coexistence. It is bounded by the hyperplanes $R_{0X}=R_{01}$, for X=2,…,N, and by a hypersurface that can be obtained as before by setting to zero the coexistence abundance of strain 1. This coexistence region persists when we allow for heterogeneous susceptibility to strain 1 as well.

Simpler versions of heterogeneous systems such as these have been shown to provide more accurate descriptions of infectious disease dynamics than their homogeneous analogues (Dwyer, Elkinton, & Buonaccorsi, 1997; Gomes et al., 2019; King, Souto‐Maior, Sartori, Maciel‐de‐Freitas, & Gomes, 2018; Langwig et al., 2017). Here, we demonstrate their capacity to support coexistence of multiple strains in a scenario where competition mediated by host immunity is maximal, as shown for two and three strains in Figure 6 and generated inductively for any natural number *N*.

Until now stabilizing mechanisms that sustain coexistence have been tied to species or strains as homogeneous static entities (Chesson, 2000; Lipsitch, Colijn, Cohen, Hanage, & Fraser, 2008). We challenge this paradigm by showing how unmeasured variation in individual fitness can stabilize coexistence across environmental conditions. Strictly, neutral models are singular in the sense that their outputs are not robust to unspecified forms of individual variation. Their use as null hypothesis should therefore be considered with care. Our arguments pertain to the interpretation of stable coexistence as evidence in support of specific niche mechanisms (Enquist, Sanderson, Weiser, & Bell, 2002; Lipsitch et al., 2008), but the rationale may be more general. Null theories should incorporate individual variation.

## 6 DISCUSSION

According to neutral theories of diversity at genetic (Kimura, 1983) and species (Hubbell, 2001) levels, the heritable variation that continually arises through mutation and migration is subject to stochastic processes that allow transient and therefore unstable coexistence of multiple genotypes or species. Stabilization of coexistence, on the other hand, can arise from specialization of genotypes or species in separate fitness peaks and ecological niches, respectively. Here, we demonstrate with two examples from bacterial systems—bacterial population growth under laboratory conditions and colonization of a host population—that nonheritable variation among individuals can stabilize coexistence in models that would otherwise be neutral. The mechanisms rely on a form of selection operating on variation in individual abilities to remain within cohorts: variation in bacterial longevity (Hartemink & Caswell, 2018; Kendall & Fox, 2002; Vaupel et al., 1979; Vaupel & Yashin, 1985), pertaining to time elapsed between cell birth and division; or variation in host susceptibility (Gomes et al., 2019; King et al., 2018; Langwig et al., 2017), referring to time since a susceptible host is born until it acquires infection. These cause cohort compositions to change in response to varying strengths of selection, providing a buffer that decreases or even hinders the effects of selection between genotypes or species and promotes coexistence.

In recent studies, intragenotypic variation has been shown to contribute to phenotypic variance to a large degree (Hashimoto et al., 2016; Jouvet et al., 2018; Kiviet et al., 2014; Shen et al., 2012; Steiner & Tuljapurkar, 2012), although the significance of these findings to the performance of neutral or adaptive theories of evolution has not been explored. While evidence is accumulating for intraspecific variation and its ecological significance (Des Roches et al., 2018; Violle et al., 2012), the literature has so far only indicated that coexistence may be weakly facilitated (Lichstein et al., 2007) or further destabilized (Hart et al., 2016) by intraspecific variation. The coexistence mechanism we describe in the context of bacterial systems is in contrast with Hart et al. (2016) in that selection is operating in our case and differs from Lichstein et al. (2007) in that selection is dynamic.

We also describe how individual variation in nonheritable fitness components is expected to bias direct measures of relative fitness between genotypes, an effect that increases with stress, resulting in inconsistent selection coefficients. We therefore propose that traditional measures of relative fitness are generated (experimentally or observationally) for several conditions across a stress gradient and a model accounting for individual variation is fitted to enable the simultaneous inference of within‐genotype variances and unbiased between‐genotype relative fitness. We consider three alternative ways to incorporate stress and obtain similar trends, although the exact formalisms should be submitted to experimental tests which are feasible in bacterial system given current technologies for high‐throughput imaging.

In summary, the importance of nonheritable variation in fitness components is now being increasingly recognized, but there are opportunities to further incorporate it into theoretical treatments and empirical tests in ecology and evolution. We illustrate some of these applications through showing impacts on genotypic fitness estimation, host use and clonal coexistence.

## CONFLICT OF INTEREST

None declared.

## AUTHOR CONTRIBUTIONS

M.G.M.G. designed the study and drafted the manuscript; all authors wrote the paper.
